# Surgery

**Published:** 1900-09

**Authors:** T. Carwardine


### SURGERY.

There is naturally much uncertainty as to what constitutes a **movable or wandering kidney**. The kidney possesses a normal movement with respiration of from one to two inches. Mansell Moullin[2] regards the failure of the kidney to ascend after a forced inspiration as a satisfactory test, even if the kidney only comes down low enough to enable its inferior extremity to be felt. In other words, the term movable kidney is a term which is relative to the movements of the diaphragm. Moullin has gone rather fully into the anatomical and theoretical considerations of the subject, and the following is an abstract of his remarks.

The kidneys are invested by the peri-renal fascia, a specialised portion of the sub-peritoneal tissue, re-inforced on the left side by certain ventral fibrous bands (Zuckerkandl). The fascia consists of anterior and posterior layers, blended externally, but separate to the inner side and below. The kidney moves freely in this capsule normally. Within this capsule, and immediately investing the kidneys, is a special fatty capsule, of a soft, delicate structure. That the outer renal capsule is not the sole factor in the retention of the organ is shown by the fact that if the abdomen be opened with the body in the erect position the kidneys fall, as if retained *in situ* by the abdominal pressure. The suggestion is thrown out that the frequency of movable kidneys on the right side may be due to a greater shallowness of the lumbar region there, owing to rotation of the tranverse processes of the vertebræ in association with the lateral deviation of the lumbar vertebræ to the left, compensatory to the deviation to the right in the dorsal region of right-handed persons. It cannot be said that the origin of movable kidney is definitely known, but if this view were correct then the left kidney should be more prone to dislocation than the right in left-handed people, an assumption which seems improbable.

A new method for fixing a movable kidney has been proposed by L. L. McArthur.[1] It consists in making a transverse incision below the last rib and stripping up the peritoneum to the middle line posteriorly. A pocket is then made in the lumbar aponeurosis behind, and the lower end of the kidney is put in the pouch so formed. Whether the kidney was designed to be put into such a pocket is doubtful, and the method strikes one as being unnecessarily severe in view of the adequate procedures in vogue.

The treatment of **enlarged prostate** is at present in an uncertain state. Dr. Wallace[2] has reviewed much of the recent literature on the subject. As regards *drainage*, the perineal route is preferred by Dandridge,[3] who thinks it safer with patients who have damaged kidneys. Sepsis plays an important part in the mortality of cystotomy. Poncet[4] quotes 117 cases, the average mortality being 25 per cent., but the mortality was three times as great in cases with septic urine as in cases in which it was aseptic. This corresponds with the statistics of Barling, who collected 169 cases of cutting operations for stone in the London hospitals. The mortality by the perineal route, in which the drainage was naturally better, was only one-third of that by the supra-pubic route. Supra-pubic drainage has, however, been much improved by the introduction of suction-drainage on the principle of the Sprengel air-pump.

The results of *vasectomy* for enlarged prostate are uncertain. In some cases the results are highly gratifying; in others, such as in carcinomatous or hard fibroid prostates, the results are disappointing. In yet another group there is some physical change in the bladder which minimises the effects of vasectomy. Reginald Harrison has considered these physical conditions from observations of prostates subsequent to vasectomy some time previously, in all of which there was evidence that the prostate had become shrunken as a result of operation,[5] although the natural powers of micturition were not regained. Harrison concludes that (*a*) vasectomy usually induces shrinkage, (*b*) which may facilitate the introduction of a catheter but not restore expulsive power, which is (*c*) associated with pouching and trabeculation of the bladder. This sacculation of the bladder is sufficient to throw the natural powers of evacuation out of gear, and cases are cited in proof of this point.

The inference drawn from these observations is that if vasectomy were resorted to at an earlier stage, *i.e.* at the stage when catheter life is usually commenced, then the expulsive

[1] *Boston M. & S. J.*, 1899, cxli. 27. [2] *Scottish M. & S. J.*, 1900, vi. 77. [3] *Ann. Surg.*, 1899, xxx. 28, 103. [4] *Bull. Acad. de Méd.*, 1898, xl. 64. [5] *Lancet*, 1900, i. 1275.

power of the bladder might be retained. Moreover, Harrison re-affirms that the sexual power persists when vasectomy alone is performed, but when the testicles are removed it is lost.

*Bottini's operation* has for some time past been revived in America, and as Dr. Willy Meyer says the operation has "come to stay," it may be well to consider the facts of the case as stated by him,[1] more particularly as the operation is applicable to cases in which vasectomy is useless. The cases, of course, require selection. Large doses of quinine are administered before and after operation. The knife is sterilized before use. The requisite electric current is obtained from the main with the help of an alternator and rheostat, or else from a storage battery with ampère-meter specially charged. In the words of Bottini, the technique requires adroitness and care in order to avoid annoying surprises. The whole operation is performed with rigid asepsis. A soft boiled catheter is partially introduced, the anterior urethra irrigated out, and then the catheter passed into the bladder. The bladder is then well irrigated and sterilized eucaine solution is injected. The current should be strong enough to turn the knife to a white heat. The instruments are finally prepared, and after five minutes the eucaine solution is withdrawn from the bladder. Then the anterior urethra is treated with eucaine solution and the instrument passed. The electric and iced-water connections are fixed, and "the prostate well hooked with the beak of the incisor exactly in the median line"—an essential point. The right forefinger is passed into the anus to test the position of the beak of the instrument. The current is turned on to the desired strength, while the operator waits about fifteen seconds for the knife to become properly heated. Then three cuts are made—one in the middle line and one in each lateral lobe, the median and most important one being made first. The suprapubic region is auscultated meanwhile, to make sure of the working of the instrument by the "sizzling" noise. This cauterization process takes ten minutes, and a final irrigation of the bladder seems unnecessary. If the patient begins to urinate voluntarily afterwards, the after-treatment is simple and the surgeon should not interfere. If there be much swelling of the gland afterwards, with retention, or if urethral fever supervenes, then difficulties arise. An anterior incision should be avoided for fear of causing inflammation of the cave of Retzius and possible death, as occurred in one case. The patient must be watched for three weeks, for hemorrhage is liable to occur during the second and third weeks. The cystoscope may first be used in all cases of doubt to determine the prostatic contour. The bladder is best injected with lotion for the purposes of the operation, for if air be used, there is probably some risk of air-embolism of the inferior vena cava.

[1] *Med. Rec.*, 1900, lvii. 705, 793.

One of the most difficult points to determine in Bottini's operation is the exact length of the incision desirable, for the surgeon works in the dark. Some idea of the length of the prostatic urethra can be gained by estimating the excessive distance a catheter must pass before a flow of urine commences, also by rectal palpation and measurement with the forefinger. When, however, the glandular elements predominate, or a pedunculated middle lobe exists, then no accurate estimate can be made. "That the occurrences here mentioned may often put the operator into a most annoying position will readily be admitted," says Meyer. Indeed, in reading Meyer's paper, one is struck by the numerous difficulties and dangers liable to present themselves, suggesting that the instrument may be a source of danger even in the best hands; and in view of these dangers and the very great pathological variety of enlarged prostates, I should require great persuasion to do Bottini's operation myself. Some of the dangers indicated are as follows:—1. Perforation of the bladder, owing to a part of the base of the bladder being caught up by the beak of the instrument. 2. Perforation of the urethra, from too long an incision with the electric knife, or an unexpectedly compressible prostate. 3. Bending of the knife. 4. Giving out of the battery. 5. Urethral fever. 6. Septicæmia and pyæmia. 7. Pulmonary embolism. 8. Absolute retention. 9. Hemorrhage; this comes on in the second or third week, when the sloughs separate; the surgeon should abstain from washing the neck of the bladder for the first few weeks, so as to lessen the danger of this. 10. Painful spasms at the neck of the bladder—a common complication.

If painful spasms and inability to urinate be apparent some time after operation, they are an indication of its incomplete performance and of the necessity to repeat the operation. Recurrence is stated to be very rare.

With regard to the advantages of the operation, the relief from the painful spasms and the increase of the patient's weight are the chief. The chronic constipation is usually relieved, the symptoms caused by cystitis are removed, and in a fair proportion of cases the turbidity of the urine disappears.

As to the indications for Bottini's operation opinions differ. Meyer thinks the operation should be done at the time when the patient is usually given the catheter to pass on himself; and that the operation is one which, according to statistics, has the lowest mortality and the highest percentage of cures.

Meyer has operated thirty times on twenty-four cases. In nine cases there was a lasting cure, and seven cases were much improved. There were two immediate and several remote deaths after the operation. Cures were about 30 per cent., improved cases rather under 30 per cent., and the mortality 16 per cent.

T. Carwardine.

